# Altered cortical activation patterns in post-stroke patients during walking with two-channel functional electrical stimulation: a functional near-infrared spectroscopy observational study

**DOI:** 10.3389/fneur.2024.1449667

**Published:** 2025-01-13

**Authors:** Sheng Xu, Shizhe Zhu, Minyao Li, Tianjiao Zhang, Qinglei Wang, Youxin Sui, Ying Shen, Kan Chaojie, Ren Zhuang, Chuan Guo, Tong Wang, Lan Zhu

**Affiliations:** ^1^Department of Rehabilitation Medicine, Changzhou Dean Hospital, Changzhou, China; ^2^Department of Rehabilitation Medicine, The First Affiliated Hospital of Nanjing Medical University, Nanjing, China; ^3^Department of Rehabilitation Medicine, The Second Affiliated Hospital of Nanjing Medical University, Nanjing, China; ^4^Nanjing Qixia District Hospital, Nanjing, China

**Keywords:** functional electrical stimulation, fNIRS, stroke, walk, cortical activation

## Abstract

Restoration of independent walking ability is the primary objective of stroke rehabilitation; however, not all patients achieve this goal due to diverse impairments in the paretic lower limb and compensatory mechanisms that lead to an asymmetrical and mechanically inefficient gait. This investigation aimed to examine alterations in cortical activation in post-stroke patients while walking with a wearable two-channel functional electrical stimulation (FES) in comparison to walking without FES. This observational study was conducted to discern distinct activation patterns in 19 stroke patients during sessions with and without FES, while using functional near-infrared spectroscopy (fNIRS) to monitor changes in blood oxygen levels. Our findings revealed only a significant reduction in ΔOxy-Hb in the contralesional pre-motor cortex (*z* = −2.803, *p* = 0.005) during the FES-on walking sessions compared to the FES-off sessions. Furthermore, all regions in the FES-on session exhibited lower ΔOxy-Hb. Conversely, no significant differences were observed in ΔDeoxy-Hb. Moreover, a significant correlation was found between decrease in cPMC and the reduced cost time of walking under FES-on condition. The fNIRS analysis revealed diminished activation in the contralesional pre-motor cortex when walking with FES, implying that FES may facilitate a more automatic gait pattern while reducing a patient’s reliance on contralesional cortical resources. The findings of this study lay the groundwork for long-term neural rehabilitation.

## 1 Introduction

Regaining independent walking ability is one of the important goals of stroke rehabilitation. However, not all stroke patients successfully regain this ability. Reports show that among inpatients undergoing rehabilitation treatment, only 39, 69, and 74% could walk independently at 3, 6, and 12 months after stroke, respectively (1). Various impairments in the affected lower limb, such as pes equinovarus, knee instability, and foot drop, contribute to the restricted walking ability (2). When effective and timely interventions are not provided, patients with stroke often resort to compensatory strategies involving the trunk and the unaffected lower limb, leading to an asymmetric and mechanically inefficient gait (3). This can result in limited mobility, an increased risk of falling, and compromised independence (4).

Promising interventions, including fitness training, high-intensity therapy, and repetitive-task training, have been identified to aid patients with stroke in recovering from an asymmetric and unstable gait (5). Among these treatments, functional electrical stimulation (FES) has been shown in previous studies to be effective in improving the clinical outcomes of patients with stroke (6–8). FES is a rehabilitation approach that involves synchronizing electrical stimulation with motor and sensory nerve fibers during functional motor tasks (9). In contrast to isolated electrical stimulation, FES utilizes rhythmic electrical stimulation targeted at specific muscles to induce functional movements that mimic voluntary contractions, thereby restoring lost functionalities (10). Recent studies utilizing neuroimaging and noninvasive brain stimulation techniques have explored the impact of FES on corticomotor function. During prolonged use of FES in rehabilitation activities, a shift in brain activity from the contralesional to the ipsilesional sensorimotor cortex has been observed when executing motor tasks involving the paretic limb (11–13). Gandolla et al. (14) reported that the combination of FES with voluntary dorsiflexion selectively heightened the sensitivity of the primary somatosensory cortex to projections from the primary motor cortex. Even a single session can lead to changes in the activation of brain areas (9, 15). Cumulatively, these studies offer compelling support for the potential efficacy of FES in fostering positive neuroplasticity.

The main goal of rehabilitation is to enhance neuroplasticity and restore motor skills to their pre-stroke levels (16). Neuroplasticity encompasses an intricate blend of both spontaneous and learning-dependent motor processes, such as restitution, substitution, and compensation (17). Research indicates that recovery from stroke heavily relies on adopting adaptive learning strategies, especially those that facilitate the reorganization of the remaining neural circuits and enable access through alternative pathways (18). Additionally, practicing task-specific training in familiar environments can boost the patient’s acquisition of similar behaviors through transferability (17, 19, 20). However, few studies have focused on specific cortical activations during FES walking, which could reveal the instantaneous effects of FES on neuroplasticity. Understanding the neural mechanisms underlying FES and observing key neuron markers could facilitate the search for a more effective way to promote recovery in patients with stroke before the onset of rehabilitation.

Among the current available neuroimaging tools, functional near-infrared spectroscopy (fNIRS) has emerged as the most suitable technique for evaluating trials involving extensive locomotion. fNIRS employs wavelengths of red light between 700 and 900 nm, capable of detecting oxyhemoglobin (Oxy-Hb) and deoxyhemoglobin (Deoxy-Hb) (21). The occurrence of heightened cortical activation, concomitant with the spontaneous elevation in blood flow within the cortices, is referred to as neurovascular coupling (22). Consequently, fNIRS can be employed to evaluate the activation level of the cerebral cortex by measuring these two parameters. Compared to functional magnetic resonance imaging (fMRI) and electroencephalography (EEG), fNIRS is less susceptible to motion artifacts and is employed for more intricate motor activities, such as walking, playing cards, among others (23). In this study, we chose Oxy-Hb and Deoxy-Hb values as the indicator to assess cortical activity.

This study aimed to investigate the changes in cortical activation of post-stroke patients during walking with wearable two-channel FES in comparison to walking without FES. Previous research has suggested that FES is primarily used for upper limb training. For example, one study reported that after 5 months of upper limb FES training, the activation of the unaffected hemisphere was significantly reduced during FES training, while the activation of the affected hemisphere was significantly increased (24). A similar effect has been observed in lower limb training, where a wearable hip-assist robot was used to produce more automatic and symmetrical gait patterns, resulting in a reduction in cortical resource costs (25). Thus, we hypothesized that patients would exhibit decreased activation of the contralesional cortex when walking with FES.

## 2 Methods

### 2.1 Participants

We recruited 19 post-stroke patients (18 male patients; mean age, 54.579 ± 11.885) in Changzhou Dean Hospital from April 11 to 28, 2023. The inclusion criteria were as follows:

1. Individuals aged 30–80 years at their first-ever hemispheric stroke.
2. The post-stroke time between 2 weeks and 12 months.
3. Brunnstrom stage of the affected lower extremity between III and V.
4. Individuals that were capable of adhering to instructions.
5. The level of the Functional Ambulation Category scale (FAC): ≥3.
6. Participants who could provide written informed consent.

The exclusion criteria were as follows:

1. Individuals suffering from progressive cerebral infarction or malignant progressive hypertension, severe visceral system diseases, malignant tumors, etc.
2. History of organic brain disease, mental disorders, and epilepsy.
3. Local skin damage, inflammation, or hyperalgesia at the site of irritation.
4. Modified Ashworth scale score of the lower extremity on the affected side: ≥3.
5. The simultaneous presence of other conditions that could influence the sensation and mobility of the lower limbs.

Body mass index, post-stroke time, stroke type, affected side, Fugl-Meyer assessment of lower extremity motor scale (FMA-LE), FAC, Berg balance scale (BBS), and 10-meter time cost (own speed with FES-on and FES-off) were collected before the experiment. The experimental protocol was approved by the Human Ethics Committee of Changzhou Dean Hospital, Changzhou, Jiangsu, China (CZDALL-2023-004) and was registered on ClinicalTrials.gov (ChiCTR2300070417). Table 1 shows the demographic information and clinical characteristics of the participants.

**Table 1:** Population characteristics.

Characteristics	Mean ± SD/n
Age (years)	54.579 ± 11.885
Gender (male/female)	18/1
BMI (kg/m^2^)	23.716 ± 2.596
Onset (day)	98.632 ± 80.747
Stroke type (ischemic/hemorrhagic)	15/4
Hemiplegic side (left/right)	9/10
Fugl-Meyer (LE, score)	26.579 ± 6.122
FAC (level)	4.053 ± 0.848
FES-off 10 m time cost (s)	10.852 ± 5.184
FES-on 10 m time cost (s)	9.611 ± 4.708
BBS (score)	48.105 ± 4.653
Quadriceps (mA)	34.111 ± 11.529
Tibialis anterior (mA)	33.389 ± 8.978

BMI, body mass index; LE, lower extremity; FAC, functional ambulation category scale; BBS, Berg balance scale.

### 2.2 Experimental design

**Figure 1:**
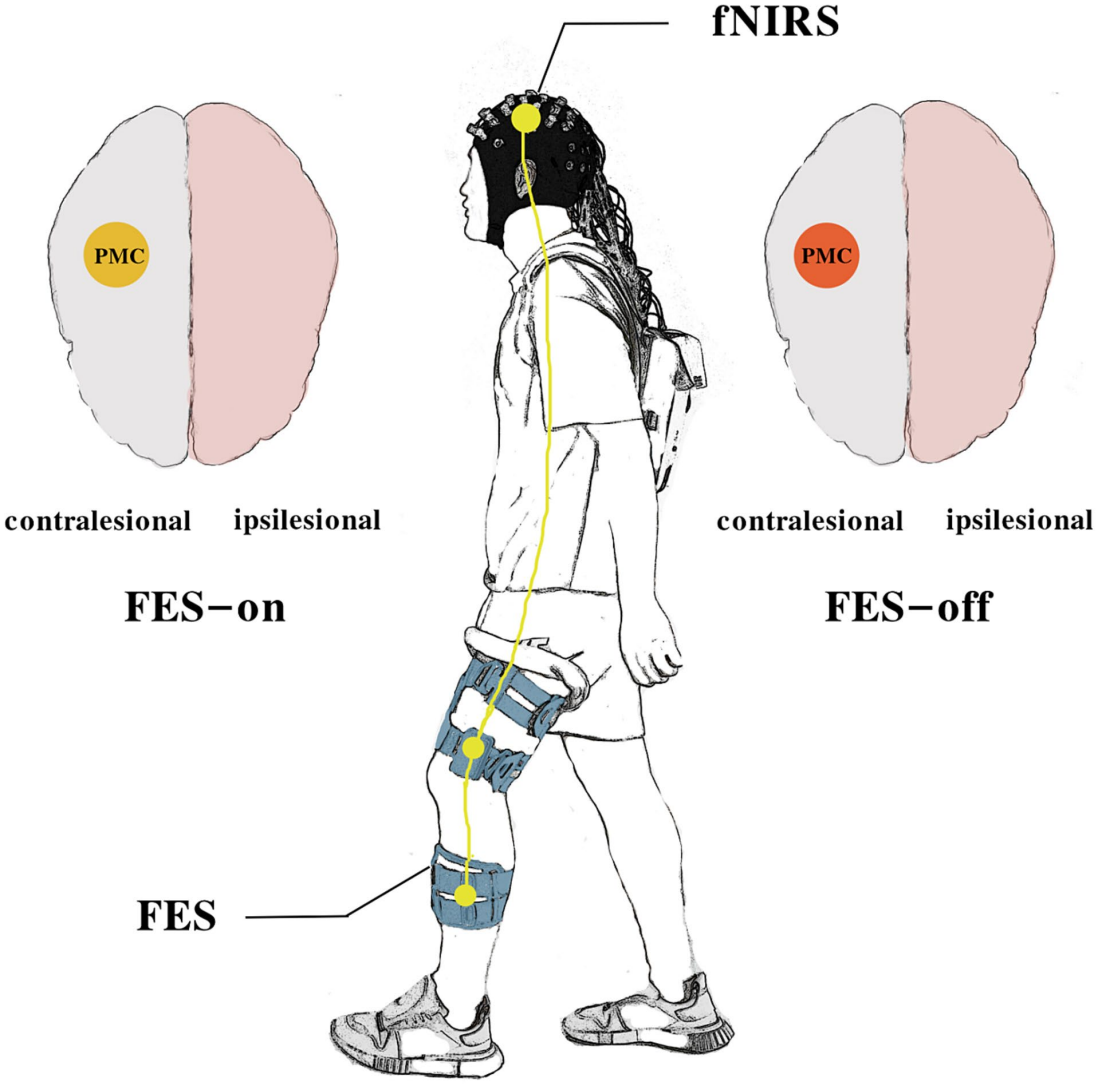
The schematic diagram of the study.

This study utilized a cross-over design wherein each participant needed to complete two different sessions of measurements. We recorded fNIRS signals simultaneously during the task. Figure 1 illustrates the schematic diagram of this trial.

### 2.3 Experiment procedures

**Figure 2:**
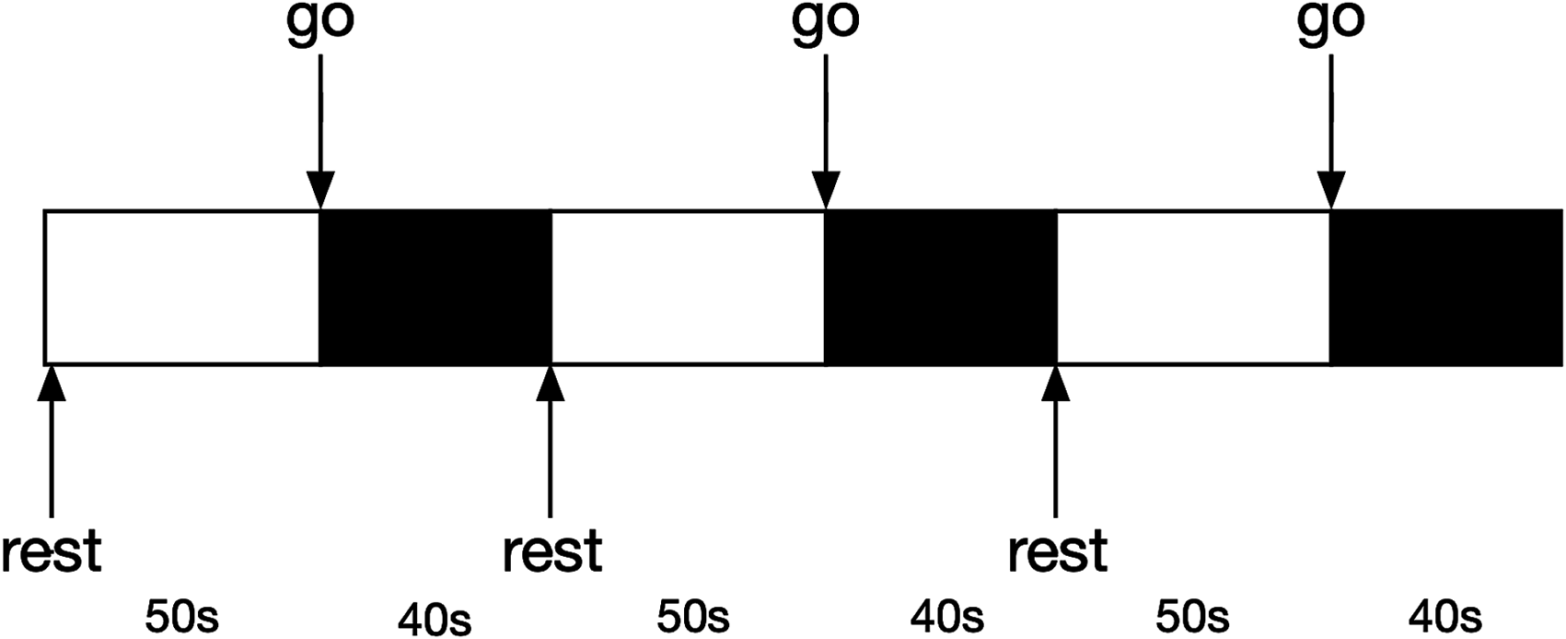
Block design diagram.

We used a block design (Figure 2) to record the changes in blood oxygen level during walking with and without FES. Notably, in the session without FES, the participants also needed to wear the device even though the equipment was powered off. The order of the two sessions was coin-random. If the coin lands heads up, data collection will start with the FES-on condition; if tails, it will start with the FES-off condition. There was a 10-min rest interval between the two sessions. All participants needed to complete both sessions. Before the onset of the experiment, the participants were equipped with the FES and fNIRS devices. A researcher adjusted the parameters to ensure they were suitable for each participant. All the participants stood upright naturally at the beginning of the experiment. After hearing the instruction “go,” the participants started to walk at their own speed and were not mandated to keep the original posture until they were told to “rest” and wait for the next trial. A total of three trials were conducted in each session. The duration of each block of walking was 40 s, with 50 s for rest. Prior to the experiment, each participant was allocated 5 min to learn and adapt to the procedures, ensuring their familiarity with the entire process. During the whole process, another researcher accompanied the participants to prevent sudden falls.

### 2.4 FES device

We used a portable FES device (YSL02P, Yasi Company, Chang Zhou, China) with electrodes that were placed at the muscle bellies of the quadriceps and tibialis anterior muscles. To ensure adequate therapeutic intensity, we selected the minimum electrical strength required to elicit visible functional and tolerated muscle contractions (Table 1) (9). We gradually increased the stimulation intensity after setting up the FES device for patients. Once visible muscle contractions occurred, we required them whether they felt the muscle contraction and if there was any discomfort. The parameters were set at a frequency of 34.5 Hz and a pulse width of 200 μs. The gait mode was designed to switch the activation order of different muscles based on a foot pressure sensor. During the stance phase, the quadriceps were activated when the sensor was pressure-activated, while during the swing phase, the switch of the tibialis anterior was turned on for the rest of the gait cycle. Due to muscle contractions induced by FES-on, blinding cannot be applied to the participants.

### 2.5 fNIRS measurement

**Figure 3:**
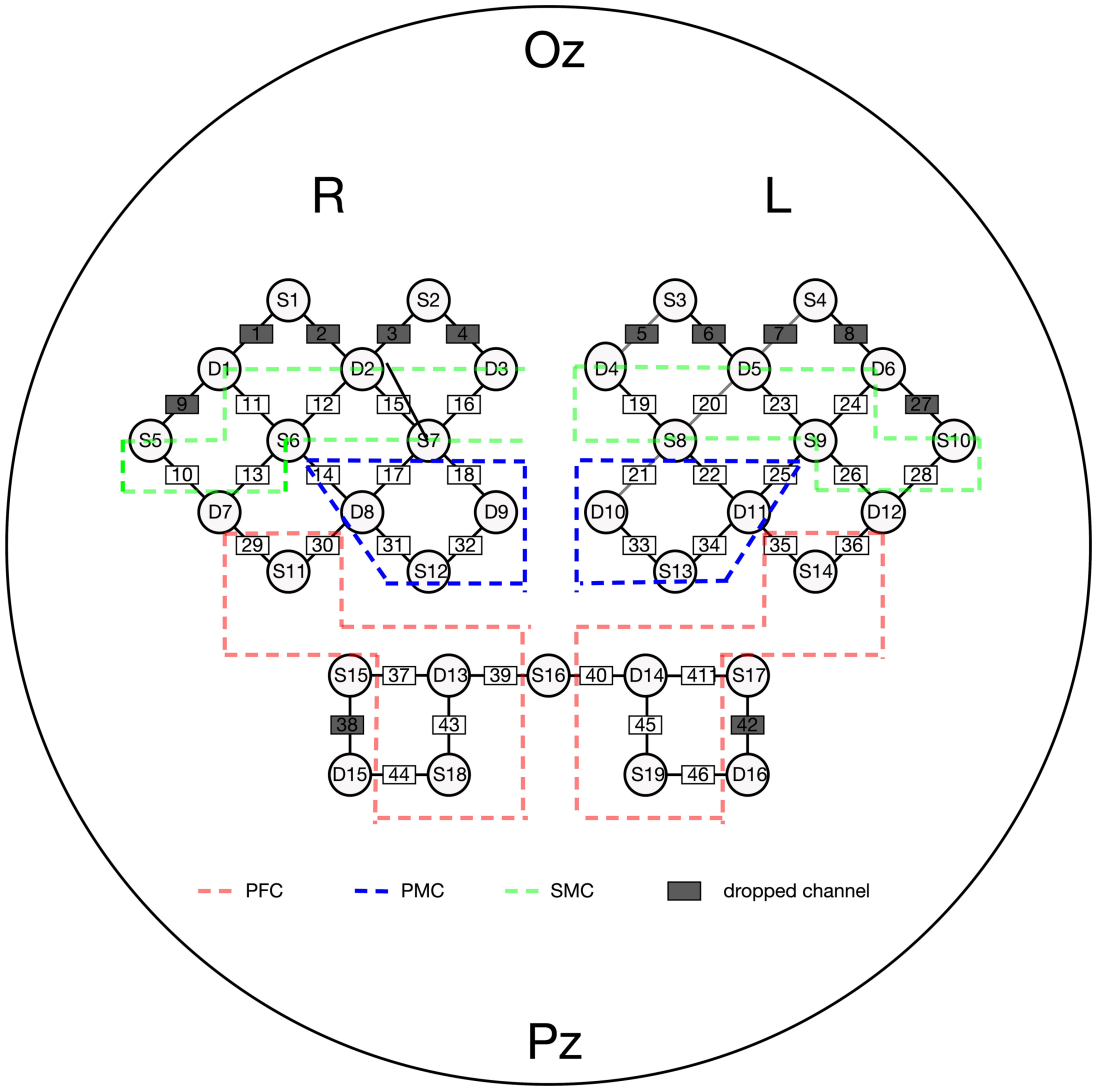
Channel attrition of fNIRS. PFC, pre-frontal cortex; PMC, pre-motor cortex; SMC, sensorimotor cortex.

The NirSmart fNIRS device (Danyang Huichuang Medical Equipment Co., Ltd., Zhenjiang, China) was used in this study. Near-infrared light (730 and 850 nm) was used to measure hemodynamic signals in the prefrontal, temporal, and parietal lobes while walking. The sampling frequency was set at 11 Hz. We positioned 45 channels (19 source optodes and 16 detector optodes) to cover the cortex. The spatial information of these optodes was based on a brain template obtained using an electromagnetic 3D digitizer device (Patriot, Polhemus, Colchester, VT, United States) (26). Channels were registered to the Montreal Neurological Institute (MNI) space and then projected to the MNI brain template. Channels were then classified according to the percentage of coverage of various Brodmann’s areas, and only channels with the highest percentage were considered representatives of functional areas. The distance between each adjacent optode was 3 cm. Given that participants with either right or left hemispheric strokes were included in the study, left hemisphere-affected participants were flipped to the right side. Thus, the right side was defined as the ipsilesional hemisphere, and the left side was defined as the contralesional hemisphere. Further details regarding channel position and the division of regions of interest (ROIs) are provided in Figure 3.

### 2.6 fNIRS data processing

We analyzed fNIRS data using the NirSpark software (HuiChuang, Zhenjiang, China) and implemented a series of preprocessing steps. Initially, the optical density signal was transformed from raw signals. Subsequently, a cubic spline interpolation algorithm (STDEV threshold = 6.0, AMP threshold = 0.5) was used to correct motion artifacts. A bandpass filter of 0.01–0.1 Hz was applied to attenuate low-frequency fluctuations and high-frequency noise, while the task-specific frequency band of the neural signals was preserved (27). Lastly, we used the modified Beer–Lambert law (path length factor = 6) to convert the filtered optical density signal to blood oxygen concentration (28). ΔOxy-Hb was chosen as the primary indicator in our analysis due to its generally superior signal-to-noise ratio compared to Deoxy-Hb (29). The “walk” marker was set as 0 s. We computed the average for the duration “0–40 s” as a block representing the hemodynamic response to the walking task, while “−2 to 0 s” served as the baseline state. We then calculated the average of the mean ΔOxy-Hb values (10^−1^ mmol/L*mm) from three repetitions to obtain an average response. The values of the channels in the regions of interest were further averaged to represent the activation of each brain region.

### 2.7 Statistical analysis

The fNIRS data were analyzed using the NirSpark software (HuiChuang, Zhenjiang, China). After obtaining the value of concentration changes, we used SPSS version 23.0 (IBM Corp., Armonk, NY, United States) for further analysis. Initially, the Shapiro–Wilk test was conducted to assess for conformity to a normal distribution. We used the Wilcoxon paired test to detect statistical significance for most of the data that did not obey the normal distribution. We also did a Spearman correlation of changes in ROIs with a significant difference (FES-on abstract FES-off) with changes in 10-meter gait speed (FES-on abstract FES-off). The significance level of the Spearman correlation is 0.05. In the result of multi-comparison, *p* values were adjusted through family-wise error correction. Because of 6 ROIs, significance levels were established at 0.008.

## 3 Results

**Figure 4:**
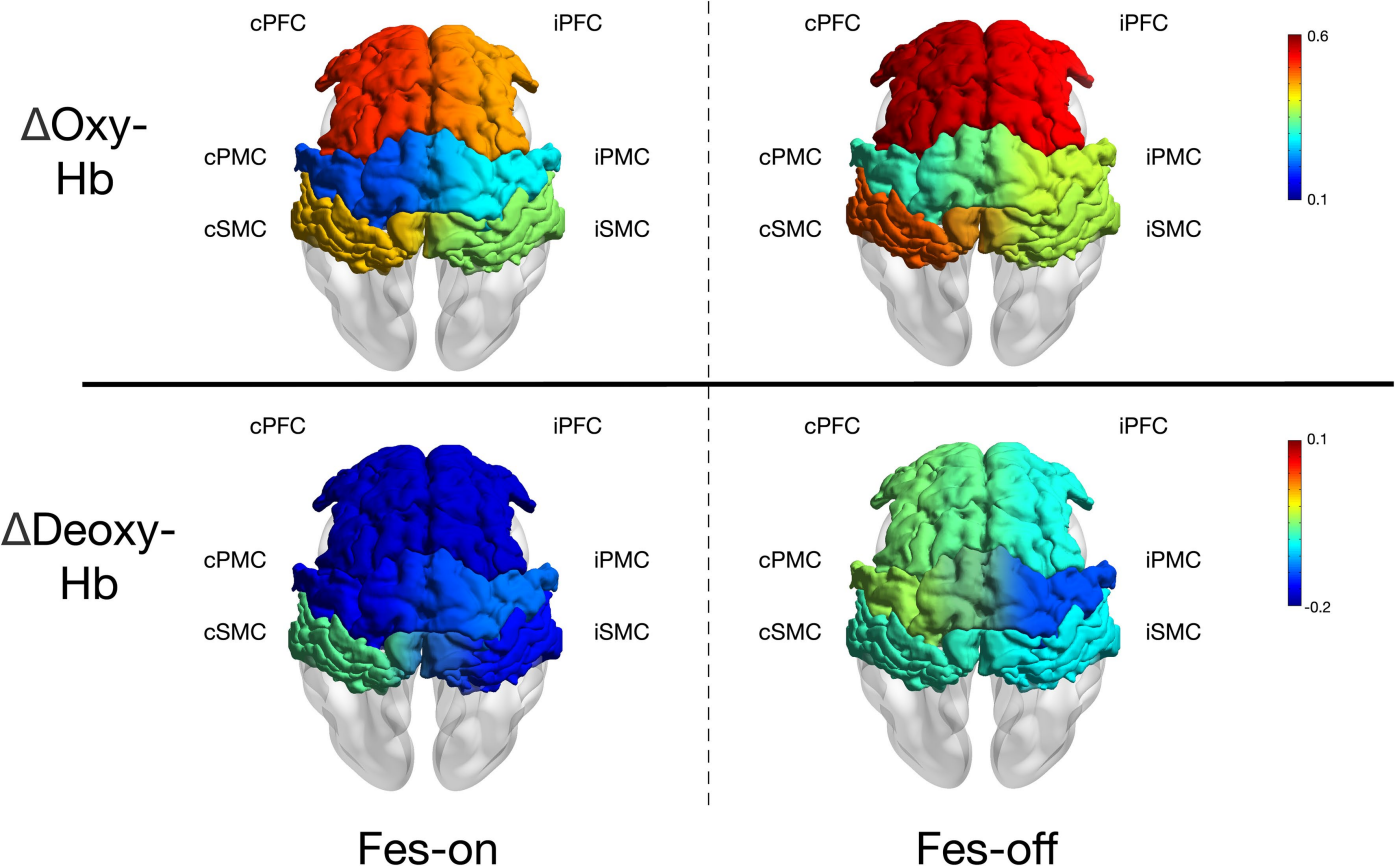
The mean ΔOxy-Hb and ΔDeoxy-Hb (10^−1^ mmol/L*mm) of each ROIs during two conditions.

The results of the study are presented in Figure 4. All participants completed the experiment without experiencing any adverse events. The ΔOxy-Hb in the contralesional pre-motor cortex cPMC (*z* = −2.803, *p* = 0.005) was found to be significantly lower during FES-on walking sessions compared to during FES-off sessions. However, no significant difference was observed in the other regions of interest (ROI). At the same time, ΔDeoxy-Hb showed no significant difference in any ROIs. Furthermore, there is a strong correlation between changes in cPMC and changes in gait speed (*ρ* = 0.509, *p* = 0.026).

## 4 Discussion

The objective of this study was to explore the impact of a wearable two-channel FES device on brain activation in patients with stroke during walking. Our results showed a significant decrease in ΔOxy-Hb in cPMC during FES-on walking sessions compared to those of FES-off walking sessions, suggesting that the use of FES may lead to a more automatic and symmetric gait pattern in patients with stroke. However, we did not observe any significant differences in the other ROIs. These findings provide evidence for the potential benefits of FES in stroke rehabilitation by modulating brain activation patterns during walking.

### 4.1 Explanation of decreased cortical activation

The application of FES led to a decrease in oxyhemoglobin (Oxy-Hb) change in the contralesional PMC during walking in patients with subacute or chronic stroke. This phenomenon may suggest that the use of the FES device requires fewer cortical resources for walking. The PMC comprises the primary motor area (PMA) and the supplementary motor area (SMA). Patients with frontal lobe dysfunction, including the PMA and SMA, often experience gait freezing due to their crucial roles in gait initiation (30). Motor programs for voluntary movements, including postural adjustment, are controlled by these regions (31). The activation of the SMA may facilitate anticipatory postural control (31, 32), while the PMA may be involved in sequencing movements activated by external stimuli (33). In a study involving bipedally walking monkeys, the injection of muscimol into the trunk/leg regions of the bilateral SMA disrupted postural control during walking, even though motor paralysis was not observed (34). On the other hand, the injection of muscimol into the dorsal PMA resulted in an inability to initiate walking following sensory guidance; interestingly, spontaneous walking remained unaffected. This suggests that the SMA may play a role in postural control, while the dorsal PMA may be specifically involved in the initiation of walking guided by sensory input.

Due to the spatial resolution limitations of fNIRS, we were unable to further divide PMC into the PMA and SMA regions. Therefore, we could only use the reduced activation of the PMC area to further explain our results. Our FES device stimulates the quadriceps femoris and tibialis anterior muscles during the gait cycle. The sequential stimulation of different muscles mimics the normal gait. When standing on the affected leg, the quadriceps femoris is activated to maintain a steady stance. During the swing phase, the tibialis anterior muscle is activated to ensure foot clearance. With the assistance of FES, the affected limb can be utilized to a greater extent during walking, thereby reducing dependence on the unaffected side. Several studies have demonstrated that long-term FES training reduces dependence on the unaffected side (12, 13, 35). Therefore, reduced dependence on the unaffected side may also be observed in cortical activation; furthermore, FES appears to play a role in postural control. Conversely, refraining from using one extremity and heavily relying on the unaffected extremity leads to significant imbalances in cortical excitation and inhibition (36, 37). In the upper extremity, a consistent observation is the reduction in corticomotor activity in the ipsilesional hemisphere paired with heightened activity in the contralesional hemisphere; moreover, several studies on the lower limb also support this fact (24, 38–40). In our study, we also found a strong association between the reduction in cPMC activation induced by FES-on and the decrease in time cost of walking triggered by FES-on. From the perspective of walking speed, this indirectly suggests that the assistance of FES could reduces the cost of resources in cPMC. However, this conclusion remains tentative, as the measurements of fNIRS and walking speed were not conducted simultaneously, which weakened the strength of this evidence. Therefore, we hypothesized that FES facilitation and less dependence on the unaffected limb could contribute to the reduced activation of the contralesional PMC.

Another noticeable phenomenon is the decreased tendency observed on both sides of every cortical region, despite the absence of significant differences between the two states. Impairments within the brain might exacerbate compensatory prefrontal recruitment for controlling walking. Existing literature indicates that the over-recruitment of the prefrontal cortex (PFC) can be attributed to various neural mechanisms, including inefficient processing, poor specificity in recruiting specialized networks, reactive recruitment in response to poor task performance, and compensatory recruitment proactively elicited to support task performance in the absence of efficient recruitment of primary brain regions or networks (41). The primary motor cortex (M1) in the sensorimotor cortex (SMC) region is known as the executor of actions. A previous investigation on monkeys demonstrated that injections of muscimol into the leg region of M1 led to localized paresis of the contralateral leg (42), indicating that PFC and SMC are involved in the entire walking process. Although few studies have investigated the neural mechanisms underlying the treatment of lower extremity functional electrical stimulation (FES) training, some studies have reported results that are similar to ours. In a study by Lee et al. (25), a group of 20 patients with chronic stroke engaged in treadmill walking at a self-selected speed while utilizing a wearable hip-assist robot, which they divided into early and late phases. Since their walking block was set at 60 s, the summary of these two phases appeared to be more comparable to those of our study. Therefore, a tendency toward decreased activation in the contralesional premotor area (PMA) during walking with the hip-assist robot was observed. The diminished activation of the SMC and supplementary motor area (SMA) may also signify more symmetric and coordinated gait patterns facilitated by rhythmic hip flexion and extension movements assisted by the robot. This pattern of reduced cortical resources in gait indicated that the robot contributed to automatic and symmetric gait patterns. As a result, with the assistance of the FES device, the activation of the PFC, PMC, and SMC showed a declining tendency. Additionally, we did not find any significant differences in Deoxy-Hb. A previous study (43) has indicated that Oxy-Hb may be more sensitive than Deoxy-Hb in detecting walking activities. This finding further supports the advantage of using Oxy-Hb to explore differences during walking.

### 4.2 The neural activation pattern of stroke gait

To better understand the results of our study, it is important to examine the activation patterns in the cortex of patients with stroke while walking or using assistive devices.

After a stroke, the cerebral cortex of patients with stroke shows increased activation of the PFC, PMA, SMA, and SMC during steady unassisted walking compared to standing; among them, most studies have shown that the PFC, PMA, and SMA are activated on both sides (41, 44–47). However, several studies have also suggested that only the contralesional PMA and SMA were significantly activated during walking (48, 49). In contrast, some studies showed that the contralesional SMC is mainly activated, with less activation on the affected side (45, 48–50). Another study (51) subdivided the walking phase and found that the contralesional PFC showed more significant activation during the entire walking process in patients with stroke; furthermore, bilateral SMC activation was increased at the beginning and acceleration phases of walking.

However, there were large discrepancies in the reports of different studies with regard to the activation pattern during assisted walking. One study showed that the activation of the PMA, SMA, and SMC in patients without the assistance of hip joint robots was lower than that observed while walking with assistance (25). Another study of exoskeleton robots pointed out that PFC activation increased when patients were assisted during walking (44). A similar study also pointed out that the activation of the bilateral SMC and the parietal central lobe of the affected side in patients with stroke was increased when robot-assisted walking was compared to weight-reducing walking; however, similar conclusions were also found in the healthy group (52). This point needs further discussion. The use of a variety of assistive devices can alter the difficulty of walking, which will lead to variations in cortical activation. In theory, facilitating the automation of walking will reduce cortical activation (41).

After relevant rehabilitation training, the PFC, PMA, SMA, and SMC have also been confirmed to have certain neural plasticity. Similar findings were found after lower limb training. A study of treadmill training found that patients showed increased activation of the ipsilesional SMC and PMC after 2 months of training (53). In another similar study, patients were divided into two groups with better and poorer walking abilities. After training, patients with better walking ability showed a trend of decreased PFC activation on the unaffected side, while patients with poorer walking ability showed a decrease in PFC activation on the affected side (54). In a 2-month weight-reduced walking training program, patients showed a significant improvement in gait after walking, which was significantly related to an increase in SMC symmetry and an increase in ipsilesional PMC.

Overall, similar to reports on the upper limb theory, several studies on the lower limb still point to the fact that recovery after a stroke often begins with compensation on the unaffected side. However, after long-term training, there will be an increase in the activation of the affected side and a gradual restoration of symmetry. This is applicable to patients with better functional recovery; however, in patients with severely impaired function, the compensation of the uninjured side is still dominant (24, 38–40).

## 5 Limitations

This study has some limitations. First, due to the limitations of fNIRS technology, we were only able to detect changes in the cortex. In the future, neuroimaging monitoring of the entire brain during naturalistic tasks is required. Second, the inability to differentiate between the PMA and SMA made our speculations uncertain, as they serve different functions, as previously mentioned. Furthermore, the low spatial resolution of our fNIRS device made it difficult to discern this difference. Although a navigation system could be a more effective tool to overcome this weakness, it is not applicable to all fNIRS devices. Third, while a previous study demonstrated the effectiveness of a two-channel FES device, it remains unclear whether more channels could have a greater impact on neuroplasticity. We hope future studies will use FES devices with more channels to investigate synchronous cortical changes during walking. Moreover, the neural control of walking is primarily governed by lower levels of the neuroaxis. Due to the limited penetration of infrared light, fNIRS can only detect cortical Oxy-Hb changes. Therefore, we are unable to observe activation changes in subcortical structures. This is a problem that current brain imaging technologies are unable to address. Disruptions in these subcortical structures can lead to compensatory recruitment of cortical resources, especially in the contralesional hemisphere (39–41). Therefore, real-time monitoring of cortical changes could help us better understand the neural mechanisms of compensation during walking. Finally, the small number of participants in our study may limit the generalization of the conclusion. At the same time, it also remains unclear whether there is a difference between the pattern in patients with subacute stroke and those with chronic stroke. Future studies could investigate this further to reveal more neural mechanisms during the stroke gait.

## 6 Conclusion

Using fNIRS technology, we designed a trial to observe synchronous cortical activation during walking with and without a wearable two-channel FES device to explore the effects of FES on neuroplasticity after stroke. Our results revealed a significant decrease in the activation of the contralesional PMC during walking with FES. Furthermore, there was a tendency toward less activation of other contralesional ROIs. These findings indicate that FES could facilitate a more automatic and symmetric gait pattern with less cortical resource cost on the contralesional side. These synchronous changes provide the basis for long-term neural rehabilitation.

## Data Availability

The original contributions presented in the study are included in the article/supplementary material, further inquiries can be directed to the corresponding authors.
